# Development of a broadly active influenza intranasal vaccine adjuvanted with self-assembled particles composed of mastoparan-7 and CpG

**DOI:** 10.3389/fimmu.2023.1103765

**Published:** 2023-03-24

**Authors:** Luis Ontiveros-Padilla, Cole J. Batty, Dylan A. Hendy, Erik S. Pena, John A. Roque, Rebeca T. Stiepel, Michael A. Carlock, Sean R. Simpson, Ted M. Ross, Soman N. Abraham, Herman F. Staats, Eric M. Bachelder, Kristy M. Ainslie

**Affiliations:** ^1^ Division of Pharmacoengineering and Molecular Pharmaceutics, Eshelman School of Pharmacy, University of North Carolina at Chapel Hill, Chapel Hill, NC, United States; ^2^ Department of Biomedical Engineering, NC State/UNC, Chapel Hill, NC, United States; ^3^ Florida Research and Innovation Center, Port Saint, Cleveland Clinic Florida, Port St. Lucie, FL, United States; ^4^ Center for Vaccines and Immunology, College of Veterinary Medicine, University of Georgia, Athens, GA, United States; ^5^ Departments of Pathology, Molecular Genetics and Microbiology and Immunology, Duke University School of Medicine, Durham, NC, United States; ^6^ Department of Pathology, School of Medicine, Duke University, Durham, NC, United States; ^7^ Duke Human Vaccines Institute, School of Medicine, Duke University, Durham, NC, United States; ^8^ Department of Microbiology and Immunology, School of Medicine, University of North Carolina at Chapel Hill, Chapel Hill, NC, United States

**Keywords:** Influenza, broadly active vaccine, intranasal, self-assembled particles, mastoparan-7, CpG

## Abstract

Currently licensed vaccine adjuvants offer limited mucosal immunity, which is needed to better combat respiratory infections such as influenza. Mast cells (MCs) are emerging as a target for a new class of mucosal vaccine adjuvants. Here, we developed and characterized a nanoparticulate adjuvant composed of an MC activator [mastoparan-7 (M7)] and a TLR ligand (CpG). This novel nanoparticle (NP) adjuvant was co-formulated with a computationally optimized broadly reactive antigen (COBRA) for hemagglutinin (HA), which is broadly reactive against influenza strains. M7 was combined at different ratios with CpG and tested for *in vitro* immune responses and cytotoxicity. We observed significantly higher cytokine production in dendritic cells and MCs with the lowest cytotoxicity at a charge-neutralizing ratio of nitrogen/phosphate = 1 for M7 and CpG. This combination formed spherical NPs approximately 200 nm in diameter with self-assembling capacity. Mice were vaccinated intranasally with COBRA HA and M7-CpG NPs in a prime–boost–boost schedule. Vaccinated mice had significantly higher antigen-specific antibody responses (IgG and IgA) in serum and mucosa compared with controls. Splenocytes from vaccinated mice had significantly increased cytokine production upon antigen recall and the presence of central and effector memory T cells in draining lymph nodes. Finally, co-immunization with NPs and COBRA HA induced influenza H3N2-specific HA inhibition antibody titers across multiple strains and partially protected mice from a challenge against an H3N2 virus. These results illustrate that the M7-CpG NP adjuvant combination can induce a protective immune response with a broadly reactive influenza antigen *via* mucosal vaccination.

## Introduction

The influenza virus represents a serious global health problem. In 2017 alone, influenza infections caused 9,459,000 hospitalizations and an estimated 145,000 deaths worldwide, with the highest mortality rate in adults older than 70 years (1–3). Influenza vaccines have been used to prevent pandemics in the past (4). However, the influenza virus strains selected to formulate seasonal vaccines can differ from the current circulating strains as a result of antigenic shift and/or drift. The difference between seasonal vaccines and circulating strains can reduce vaccine efficacy significantly, highlighting the need to provide more broadly reactive vaccines (5). To produce a broadly reactive influenza vaccine, a computationally optimized broadly reactive antigen (COBRA) was developed by obtaining iterative consensus sequences of the hemagglutinin (HA) antigen from decades of circulating influenza strains. COBRA-based vaccines can elicit a broadly reactive immune response and protection against existing and emergent strains (6–8). Recombinantly produced COBRA HA-based vaccines, like most subunit antigens, need to be adjuvanted to initiate a protective response. A majority of the currently licensed adjuvants are direct innate immune activators, such as CpG or monophosphoryl lipid A (MPLA), which can bind and activate innate immune receptors toll-like receptor 9 (TLR 9) and TLR 4, respectively. However, a new group of molecules are being studied for their capacity to target specific types of innate immune cells, especially mast cells (MCs) (9, 10).

MCs have been implicated in rapid, robust, and sustained inflammatory responses in the body (e.g., allergic hypersensitivity), but also have roles in immunity against infectious pathogens (11, 12). Upon exposure to bacteria, MCs regulate recruitment of immune cells to local draining lymph nodes (dLNs) (13) in a manner dependent on MC production of TNFα (14–16). Moreover, MC-derived mediators have been shown to be implicated in the alteration of the lymph flow and promote the antigen entry to the dLN (17). These mediators (cytokines, histamine, and proteases) are also involved in the modification of the dLN microarchitecture (i.e., hyperplasia), allowing the accumulation of peripheral lymphocytes (18, 19).

When formulated with an intranasally (IN) administered anthrax protective antigen, MC activator c48/80 has been shown to provide a safe and effective adjuvant effect comparable with the gold standard mucosal vaccine adjuvant, cholera toxin (14). Additionally, intradermal (ID) injection of c48/80 does not generate IgE, which is an indicator of hypersensitivity. By contrast, cholera toxin generates significantly greater IgE responses (20), mainly because its receptor (GM1) is expressed in a large variety of cells involved in hypersensitivity (eosinophils, basophils, and MCs) (21). Even in an attempt to trigger anaphylaxis by injecting anthrax protective antigen intraperitoneally into mice that were IN immunized with c48/80 and anthrax protective antigen, no signs of anaphylaxis or distress were reported (14). Overall, regardless of route, MC activators have been shown to be safe and effective. Importantly, despite the role of MCs in allergic-type reactions, the use of MC activators as vaccine adjuvants in preclinical models is safe without signs of allergic reactions or elevated antigen-specific IgE (14, 20, 22). Moreover, polymyxins (which are also MC activators) are FDA-approved compounds that have been shown to be safe in humans, including aerosolized administrations at optimal doses.

Currently licensed adjuvants are primarily designed for parenteral immunization routes (23), whereas mucosal adjuvants are needed to best combat respiratory pathogens, such as influenza. Both small-molecule and peptide-based MC agonists have been reported to degranulate MCs and induce the release of important inflammatory mediators, such as pro-inflammatory cytokines (10, 24–26). One peptide-based MC agonist is mastoparan-7 (M7). M7 is a cationic peptide derived from wasp venom with strong adjuvant activity upon IN administration. M7 can activate G-protein signaling *via* MC membrane receptor MrgprX2, resulting in the degranulation and activation of MCs (10, 27, 28). When delivered with an antigen, M7 can induce neutralizing antibody titers against viral antigens and improve the protection against viral challenges (29, 30).

We combined MC agonist peptide M7 with TLR 9 ligand CpG to generate an IN adjuvant system (M7-CpG) with the capacity to induce a potent inflammatory response in the respiratory mucosa. CpG is an adjuvant composed of multiple synthetic oligodeoxynucleotides (rich in unmethylated cytosine-guanine motifs) with the capacity to induce expression of proinflammatory cytokines and costimulatory molecules in multiple cells, including dendritic cells (DCs), macrophages, and total leukocytes (31–33). CpG-adjuvanted vaccines generate long-lasting antibody responses as well as CD4^+^ and CD8^+^ T-cell responses against co-immunized antigens like OVA (34). Importantly, CpG promotes type-I interferon release and Th1 responses against different co-administered antigens, both of which are important in generating effective antiviral responses (35, 36).

In this study, novel adjuvant system M7-CpG was co-administered with COBRA HA protein to generate an IN broadly reactive vaccine against different influenza strains. Humoral, cellular, and protective responses were evaluated in a mouse model.

## Materials and methods

### M7-CpG NPs fabrication and characterization

M7 peptide (10 nmol = 14.22 µg) (Biomatik, Wilmington, DE, USA) from a stock of 1 mg/ml of M7 in PBS was combined at different nitrogen/phosphate (N/P) ratios with CpG ODN 1826 (Invivogen, San Diego, CA, USA) (20 µg for N/P = 0.5, 10 µg for N/P = 1, 5 µg for N/P = 2, and 3.2 µg for N/P = 3) from a stock solution of 1 mg/ml of CpG in PBS (Sigma-Aldrich, St. Louis, MO, USA) and sonicated in a water bath at 100% amplitude (Branson, Danbury, CT, USA) for 20 min and at room temperature (RT). Particle size at N/P = 1 was determined using dynamic light scattering (DLS), and particle surface charge through electrophoretic light scattering (ELS) zeta potential (Brookhaven, Holtsville, NY, USA). Particle shape and size were also evaluated using transmission electron microscopy (TEM) (Thermo Scientific Talos F200X) and scanning electron microscopy (SEM) (Hitachi S-4700, Japan). The endotoxin content of M7, CpG, or the combination was analyzed *via* a limulus amoebocyte lysate (LAL) endotoxin assay (Thermo Fisher Scientific, Waltham, MA, USA). All samples had undetectable endotoxin levels (<0.1 EU/ml at 1 mg/ml).

### Storage stability

M7-CpG NPs were stored in triplicate at −20°C, 4°C, or 20°C (RT) for 15 days and particle stability was measured on days 0, 5, 10, and 15 by DLS.

### *In vitro* cytotoxicity and innate immune response assays

Mouse DC line DC2.4 (ATCC, Manassas, VA, USA) and mouse lung epithelial cell line LA-4 (ATCC) were cultured in RPMI 1640 medium (Corning, Corning, NY) supplemented with 10% fetal bovine serum (VWR, Radnor, PA, USA) and 10 U/ml penicillin–streptomycin (Thermo). Mouse MC line MC/9 (ATCC) was cultured in DMEM medium (Sigma-Aldrich) supplemented with 2 mM L-glutamine, 0.05 mM 2-mercaptoethanol, 10% Rat T-STIM (BD, Franklin Lakes, NJ, USA), and 10% fetal bovine serum (VWR). All cell lines were stimulated with M7, CpG ODN 1826, or M7-CpG NPs. Samples of cell culture supernatants were taken 24 h and 48 h post-stimulation for a colorimetric lactate dehydrogenase (LDH) release cytotoxicity assay (Thermo). The percentage of LDH release was normalized to a positive control (cells treated with Thermo 1× lysis buffer for 10 min) at 100% and to a negative control (no-treatment cells) at 0%. Cytokine ELISAs were also performed on supernatants according to the manufacturer’s protocols to assess TNF-α and IL-6 production (Thermo).

MC degranulation levels were measured by β-hexosaminidase release. Briefly, 5 × 10^5^ MCs were cultured in Tyrode’s buffer (137mM NaCl, 2.7 mM KCl, 1.8 mM CaCl_2_, 1 mM MgCl_2_, 0.2 mM Na_2_HPO_4_, 12 mM NaHCO_3_, and 5.5 mM D-glucose) in a 96-well plate. Cells were treated with M7, CpG, M7-CpG NPs, and Triton X-100 (Sigma-Aldrich) at 37°C and supernatants were collected after 30 min. A total of 30 µl of supernatant from each well was incubated with 10 µl of p-nitrophenyl-N-Acetyl-β-D-glucosaminidase (NAG) substrate solution [3.4 mg/ml NAG (Sigma-Aldrich) in citrate buffer (pH 4.5)] for 1 h at 37°C and then 100 µl of carbonate buffer pH 10 was added to develop a colored substrate that was measured at 405 nm. Degranulation percentages were obtained by subtracting background absorbance from non-stimulated cells and normalizing 100% degranulation to cells stimulated with Triton X-100.

### COBRA HA

The full-length sequence of HA from different human H3N2 viruses (from May 2013 to April 2016) was collected from the Global Initiative on Sharing Avian Influenza Data (GISAID) EpiFlu online database and used to create a consensus sequence based on a COBRA methodology previously described (8, 37).

### Immunization

C57BL6/J female mice (8–10 weeks old; Jackson Labs) (*n* = 10) were immunized IN in each nostril (7.5 µl per nostril) on days 0, 21, and 35 with PBS, COBRA J4 HA antigen (J4) (3 µg), M7 (10 nmol) + J4 (3 µg), CpG (10 µg) + J4 (3 µg), or M7-CpG NPs (10 nmol + 10 µg, respectively) + J4 (3 µg). All animal experiments were performed in accordance with the UNC Institutional Animal Care and Use Committee (IACUC). Submandibular blood and fecal samples were collected on days 14, 28, and 42. Mice were sacrificed on day 42, and bronchoalveolar lavages (BALs), nasal washes, and spleens were collected. To perform BAL collection, a 22Gx1” catheter (Thermo) was inserted in each mouse’s trachea and 1 ml of 1× PBS supplemented with protease inhibitor cocktail (Roche) (1 tablet/10 ml) and Triton X-100 (Sigma-Aldrich) (0.01% v/v) was flushed three times towards the lungs. To perform nasal washes, the catheter was re-inserted in the trachea and fresh solution was flushed towards the nose. Wash fluids were collected in sterile 1.5-ml microcentrifuge tubes (Sigma-Aldrich) (38, 39). Spleens were harvested and processed into single-cell suspensions for antigen recall.

### Mouse sample processing for antibody titers

Fecal samples were snap frozen and stored at −80°C until evaluation of antibody titers. Prior to analysis, fecal samples were defrosted, diluted to 10 mg/100 μl in protein extraction buffer [10% goat serum (MP Biomedical, Santa Ana, CA, USA) in PBS], and vortexed for 20 min or until fully dispersed. Fecal samples were centrifuged for 10 min at 13,000 × *g*, and supernatants were collected for ELISAs (40). Sera were obtained by centrifugating the collected blood for 10 min at 3,000 × *g* (Greiner Bio-One, Monroe, NC) then stored at −20°C. BAL and nasal wash samples were stored at −20°C. For the evaluation of antibody titers, all frozen samples were defrosted and used directly for ELISAs.

### Antibody titers

High-binding flat bottom 384-well plates (Greiner Bio-One) were coated overnight with 100 ng/ml of COBRA J4 HA in PBS at 4°C, washed 3× with 0.05% Tween 20 in PBS (PBST) *via* a plate washer (Biotek ELX405) and then blocked for 2 h [3% non-fat instant milk in PBS (blocking buffer) at RT]. Plates were then washed 3× (PBST), and samples (serum, BAL, or fecal) diluted 1:100 in 100 μl of blocking buffer were added. Five times serial dilutions were performed, added to the plates, incubated for 1 h (RT), and washed 3× (PBST). The appropriate secondary antibodies [goat anti-mouse IgG-HRP, goat anti-mouse IgM-HRP, goat anti-mouse IgG1-HRP, goat anti-mouse IgG2c-HRP, goat anti-mouse IgA, or goat anti-mouse IgE (Southern Biotech)] were diluted in blocking buffer to the highest dilution recommended by the manufacturer and added to the plates. Plates were incubated for 2 h (RT), washed 5× (PBST), and treated with tetramethylbenzidine (TMB) one-component substrate (Southern Biotech) until developed. The reaction was stopped with 25 μl of 2 N sulfuric acid and absorbance was read at 450 nm in a plate reader (Biotek ELX405, Agilent). Endpoint titers were defined as the lowest serum dilution at which the absorbance was three standard deviations above the average negative control (41).

### ELISPOT

MultiScreen-IP 0.45-µm filter 96-well plates (Sigma-Aldrich) were pre-wetted for 1 min with 70% ethanol and coated overnight at 4°C with 100 µl of anti-IL-2 or anti-IFN-γ capture antibody (BD) diluted in PBS according to the manufacturer’s protocol. Plates were blocked for 2 h with 10% FBS in RPMI 1640 medium. Splenocyte suspensions were obtained from mouse spleens according to a previous protocol (42), and 10^6^ splenocytes from immunized mice were plated and stimulated with 100 μl of COBRA J4 HA (10 μg/ml in RMPI 1640 complete medium). Plates were incubated for 36 h at 37°C and washed 3× (PBST). Then, 100 µl of anti-IL-2 or anti-IFN-γ capture antibody (BD) diluted in FBS 10% in PBS was added to the plates according to the manufacturer’s protocol. Plates were incubated for 2 h (RT) and washed 3× (PBST). Streptavidin-HRP (100 µl; BD) diluted in FBS 10% in PBS was added to the plates according to the manufacturer’s protocol, and plates were incubated for 1 h (RT) before being washed 4× with PBST then 1× with PBS. Solution [100 µl of 3-amino-9-ethylcarbazole (AEC) substrate + chromogen (BD)] was added until spots were observable. Plates were then washed 2× (PBS) and dried (RT 48 h). Spots were counted using an ImmunoSpot plate reader (CTL).

### Antigen recall

Splenocytes (1 × 10^6^) from mice were plated in duplicate in a flat-bottom tissue culture 96-well plate (Corning), stimulated with 100 μl of COBRA J4 HA (10 μg/ml in RPMI 1640 complete medium), and incubated for 36 h at 37°C. Cytokine ELISAs were performed with culture supernatants to evaluate IFN-γ, IL-4, and IL-17A (Thermo).

### CD4+ T-cell memory phenotype

Cells from the dLN were plated similarly to splenocytes for antigen recall. Cells were stained for flow cytometry with efluor 506 viability dye (Thermo), anti-mouse CD3-AF488 (BioLegend, San Diego, CA, USA), anti-mouse CD4-BV421 (BD), anti-mouse CD44-APC (BioLegend), and anti-mouse CD62L-BV785 (BD). Cells were analyzed with an Attune NxT flow cytometer (Thermo).

### Hemagglutinin inhibition titers

Sera were diluted threefold with receptor destroying enzyme (RDE) (Denka Seiken, Japan). After an overnight incubation at 37°C, the RDE was inactivated at 56°C for 30 min. After cooling to RT, sterile PBS was added to give a final serum dilution of 1:10. Samples were stored at 4°C until use. RDE-treated serum (50 µl/well) was then added to 96-well V-bottom microtiter plates (column 1) and 25 µl was twofold serially diluted in PBS across the plate. Next, 25 µl/well of virus, titrated to 8 HAU/50 µl per well, was added for a 20-min incubation at RT. Then, 1% horse red blood cells (HRBC) (Lampire Biologicals) in PBS was added. The plates were covered, agitated to mix, and then incubated at RT for 1 h. Hemagglutinin inhibition (HAI) was calculated as the reciprocal dilution for the last well, which did not contain agglutinated HRBCs (43).

### Viral challenge

C57BL6/J female mice (8–10 weeks old; Jackson Labs) (*n* = 10) were immunized as stated above and challenged IN on day 56 post-immunization with 1,000 PFU (50 µl) of the mouse-adapted virus A/Hong Kong/1/68 (BEI resources). Mice were weighed and scored daily for 14 days, according to previous reports (44, 45); a 20% loss of body weight or a moribund body condition (clinical score = 6) were used as survival endpoints. Mice were clinically scored during the challenge according to previously reported criteria (44, 45).

### Statistics

All *in vitro* and *in vivo* data were analyzed using a parametric test, except survival data. Parametric analysis allows the comparison between different experimental groups (5, 46, 47). All *in vitro* studies, mouse weights, and clinical scores were analyzed with a two-way ANOVA test with post-hoc Tukey’s multiple comparisons. Antibody titers, HAI titers, and T-cell response studies were analyzed with a one-way ANOVA test with post-hoc Tukey’s multiple comparisons. Survival studies were analyzed using Kaplan–Meier survival statistics in Prism. All tests were performed using the software GraphPad Prism 6^®^ adjusting *p*-values for multiple comparisons.

## Results

### M7 and CpG nanoparticle complexation and immune response in dendritic and mast cells

**Figure 1:**
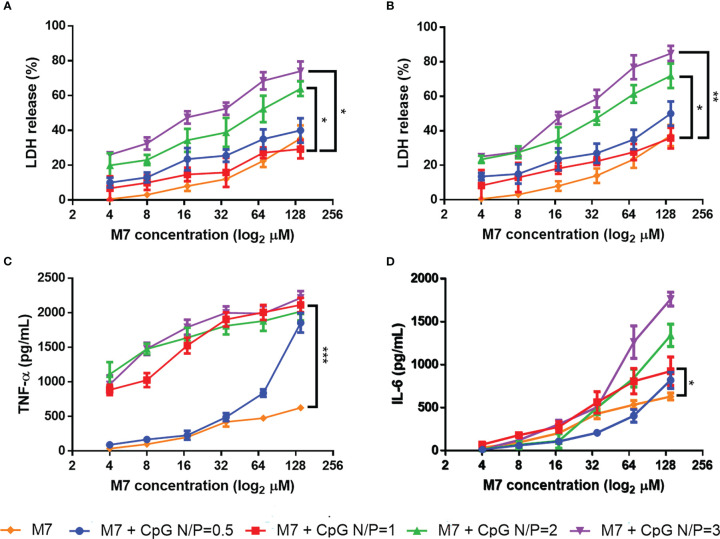
M7 and CpG combined at an N/P ratio = 1 induce the lowest cytotoxicity and the best proinflammatory cytokine release. M7 was mixed at different nitrogen/phosphate (N/P) ratios with CpG and cultured with DC2.4 cells at different dilutions. Cytotoxicity was measured through LDH release at **(A)** 24 h and **(B)** 48 h post-stimulation. **(C)** TNF-α and **(D)** IL-6 expression in culture supernatants were evaluated at 48 h post-stimulation (*n* = 6, two independent experiments). Data are presented as mean ± range. Significant differences were determined using a two-way ANOVA test with Tukey’s multiple comparison (*p-value ≤ 0.05; *p-value ≤ 0.01; ***p-value ≤ 0.005).

M7 and CpG were first assessed as a mixture and evaluated for innate immune activity. DC2.4 cells were treated with M7/CpG mixtures at different N/P ratios (0.5, 1, 2, and 3), and cytotoxicity was evaluated by LDH assay. Although all M7/CpG mixtures induced some cytotoxicity at high concentrations, N/P ratios of 1 and 0.5 exhibited the lowest cytotoxicity. Because the N/P = 1 mixture exhibited reduced cytotoxicity compared with N/P = 0.5 (**Figures 1A, B**), the cytotoxicity of CpG alone was also evaluated and it illustrated an approximately 40% cytotoxicity at the highest concentration after 48 h (**Figure S4**). To determine whether CpG/M7 mixtures exhibited adjuvant activity, the release of the proinflammatory cytokines TNF-α and IL-6 was measured. M7/CpG mixtures generally stimulated greater secretion of both IL-6 and TNF- α relative to soluble M7 alone, with the most robust stimulation occurring at N/P ratios ≥ 1 (**Figures 1C, D**; **Table S1**). Although there were no significant differences in cytokine secretion for cells cultured with N/P ratios of 1, 2, or 3, we decided to further study the combination of M7 and CpG at N/P ratio = 1 because it induced the highest levels of proinflammatory cytokine secretion and had the lowest cytotoxicity among combinations.

**Figure 2:**
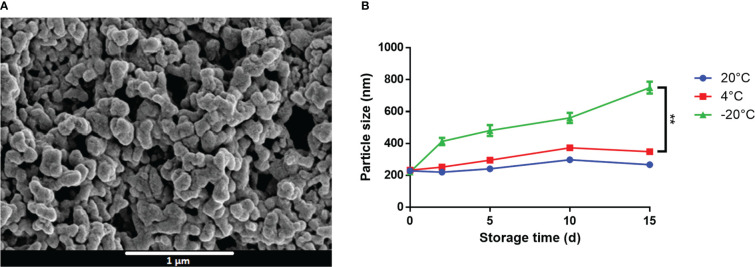
M7 and CpG form stable self-assembled NPs. **(A)** SEM micrographs of M7-CpG NPs (N/P ratio = 1) and **(B)** DLS particle size of M7-CpG NPs (N/P ratio = 1) over time at different temperatures. Data are presented as mean ± standard deviation (*n* = 3). Significant differences were determined using a two-way ANOVA test with Tukey’s multiple comparison (**p-value ≤ 0.01).

Owing to their opposing charges, we hypothesized that CpG and M7 might interact electrostatically to form a macromolecular complex. SEM micrographs of M7 and CpG at a N/P ratio of 1 detail the formation of spherical particles with an average diameter of 200 nm, herein referred to as M7-CpG NPs (**Figure 2A**). The size and shape were confirmed with TEM (**Figure S1**). Surprisingly, the zeta potential of the M7-CpG NPs (N/P = 1) revealed a negative surface charge, despite an equimolar concentration of positive and negative charges (**Table S2**). This may suggest localization of CpG to the particle surface due to its large size. Particle stability was assessed by storing M7-CpG NPs at different temperatures (−20, 4, or 20°C) for 15 days. Average particle size and polydispersity index (PDI) remained consistent for samples stored at 4 or 20°C (**Figure 2B**; **Table S3**), suggesting good stability even at relatively high temperatures.

**Figure 3:**
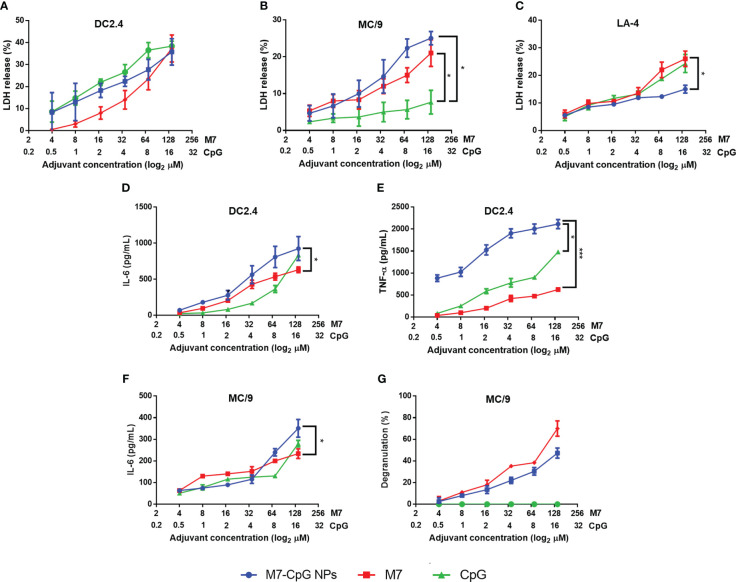
M7-CpG NPs decrease cytotoxicity and increase innate immune response *in vitro*. **(A)** DC2.4 cells, **(B)** MC/9 cells, and **(C)** LA-4 cells were stimulated with M7, CpG, or M7-CpG NPs and cytotoxicity was measured by LDH release at 48 h post-stimulation. **(D)** IL-6 and **(E)** TNF-α expression was evaluated in culture supernatants from DC2.4 cells stimulated with the same conditions at 48 h post-stimulation. **(F)** IL-6 expression and **(G)** degranulation levels in supernatants were evaluated in MC/9 cells stimulated with the same conditions at 30 min and 48 h post-stimulation, respectively. Data are presented as mean ± range (*n* = 6, two independent experiments). Significant differences were determined using a two-way ANOVA test with Tukey’s multiple comparison (*p ≤ 0.05; ***p ≤ 0.005).

In DC and MC cultures, M7-CpG NPs were not significantly more cytotoxic than the most cytotoxic individual adjuvant (**Figures 3A, B**). However, in non-phagocytic LA-4 (lung epithelial) cells, NPs were less cytotoxic than M7 or CpG alone (**Figure 3C**). For the DCs, an increase in the secretion of soluble TNF-α and IL-6 was observed with NP treatment (**Figures 3D, E**). MCs illustrated similar levels of degranulation upon NP or M7 treatment, but CpG alone did not induce detectable degranulation. The expression of IL-6 in MCs was greater in NP cultured cells compared with MCs cultured with M7 or CpG alone (**Figures 3F, G**).

### M7-CpG NPs *in vivo* vaccination studies

**Figure 4:**
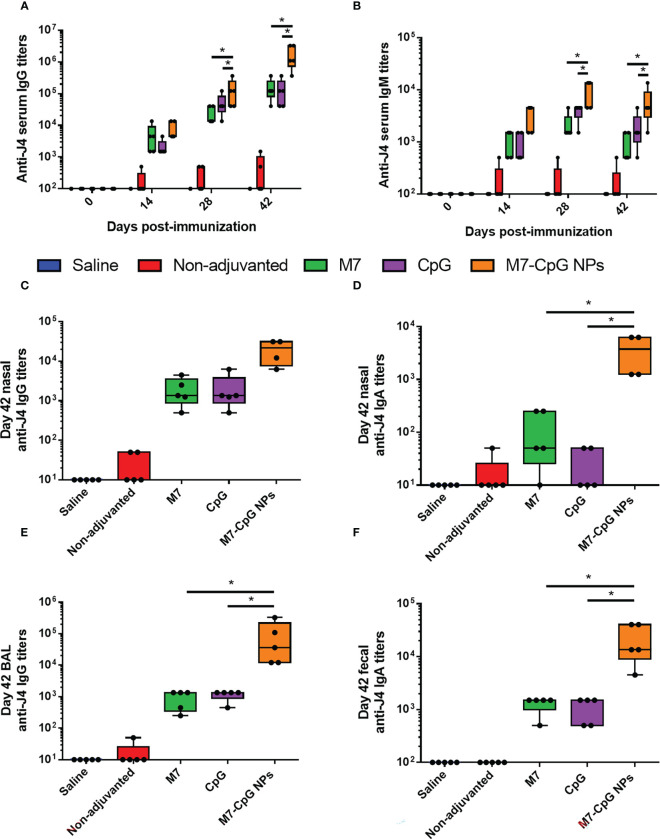
M7-CpG NPs improve the serum and mucosal antibody response against the COBRA influenza antigen J4. C57BL/6 mice were intranasally immunized with saline, non-adjuvanted J4, M7 + J4, CpG + J4, or M7-CpG NPs + J4 on days 0, 21, and 35. **(A)** J4-specific IgM and **(B)** total IgG titers on days 0, 14, 28, and 42 post-immunization. **(C)** J4-specific total IgG and **(D)** IgA titers in nasal wash on day 42 post-immunization. **(E)** Total IgG titers in bronchoalveolar lavage (BAL) and **(F)** IgA titers in feces on day 42 post-immunization. Data are presented as mean ± range (*n* = 5) (representative plots of two independent experiments). Significant differences were determined using a one-way ANOVA test with Tukey’s multiple comparison on the data shown (*p ≤ 0.05).

Mice were vaccinated IN with M7-CpG NPs and the COBRA HA antigen (J4) in a prime–boost–boost schedule on days 0, 21, and 35. An approximately 2-fold increase in IgG titers against J4 was found in the serum of mice vaccinated with NPs, compared with single adjuvanted mice on day 28 post-immunization, and on day 42, an approximately 10-fold increase was found. For IgM titers, a 10-fold increase was observed on days 28 and 42 post-immunization (**Figures 4A, B**). J4-specific IgG1 and IgG2c antibody titers were measured to characterize the Th1 and Th2 responses induced by our NPs. Serum IgG1 and IgG2c, and nasal IgG and IgA, were all higher in samples from mice vaccinated with NPs than mice vaccinated with the separated adjuvants (**Figures S2**, **4C, D**). A similar phenomenon was observed for BAL samples, in which the J4-specific IgG response was enhanced in NP-vaccinated mice (**Figure 4E**). Finally, higher titers of J4-specific IgA were present in feces from mice vaccinated with M7-CpG NPs (**Figure 4F**).

**Figure 5:**
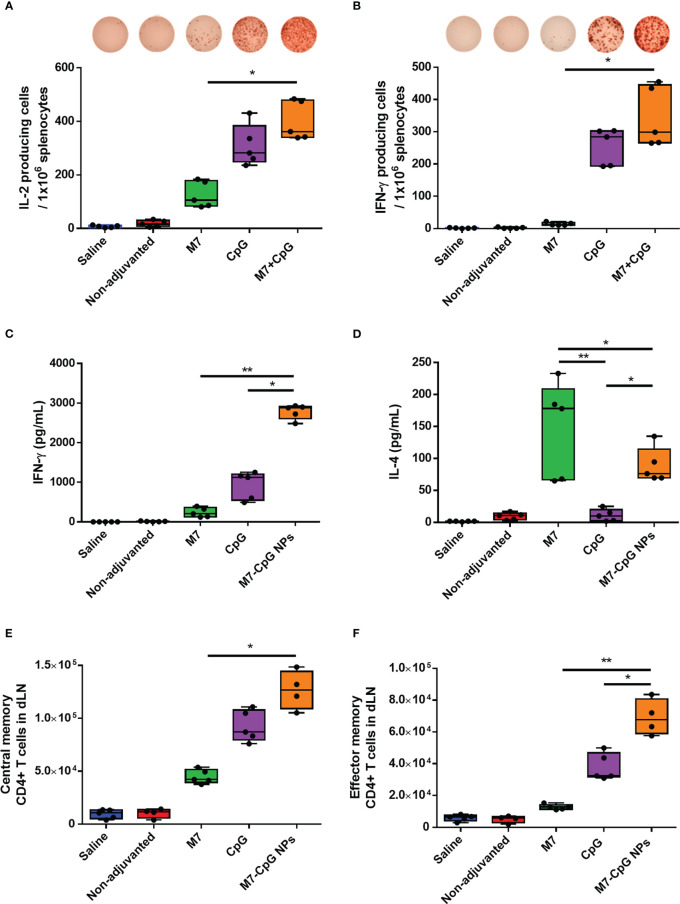
M7-CpG NPs potentiate and polarize the T-cell response against the COBRA influenza antigen J4. C57BL/6 mice were intranasally immunized with saline, non-adjuvanted J4, M7 + J4, CpG + J4, or M7-CpG NPs + J4 as previously described. Spleens and draining lymph nodes (dLNs) were harvested on day 42 post-immunization. ELISPOT counts for **(A)** IL-2 and **(B)** IFN-γ from antigen-recalled splenocytes. **(C)** IFN-γ and **(D)** IL-4 release was evaluated in supernatants from antigen-recalled splenocytes. Total number of **(E)** central memory and **(F)** effector memory CD4+ T cells in dLN. Data are presented as mean ± range (*n* = 5) (representative plots of two independent experiments). Significant differences were determined using a one-way ANOVA test with Tukey’s multiple comparison on the data shown (*p ≤ 0.05; **p ≤ 0.01).

To examine the cellular response to vaccination, spleens were harvested 42 days post-immunization and used to perform cytokine specific ELISPOTs and ELISAs. After antigen stimulation, greater levels of IL-2 and IFN-γ-expressing cells were observed in the spleens of M7-CpG NP-vaccinated mice compared with the M7 group, and results trended towards higher levels in mice vaccinated with M7-CpG NPs compared with the CpG group (**Figures 5A, B**). IFN-γ levels were found to be at least 3-fold higher in mice vaccinated with M7-CpG NPs compared with the separate adjuvant groups (**Figure 5C**). On the other hand, IL-4 levels were greater in the M7 adjuvanted group than in the M7-CpG NP adjuvanted group (**Figure 5D**). Levels of IL-2 and IL-17 were also measured in culture supernatants from J4-stimulated splenocytes. The highest expression of IL-2 was seen in splenocytes from M7-CpG NP-treated mice, while the levels of IL-17 were similar between mice immunized with NPs or M7 alone (**Figures S3A, B**). In the dLNs, greater frequencies of central and effector memory CD4+ T cells were observed from M7-CpG NP-vaccinated mice compared with other vaccination groups, as is noticeable in representative dot plots (**Figure S3C**). The total count of central memory CD4+ T cells in dLNs was found to be increased in M7-CpG NP-vaccinated mice compared with M7-vaccinated mice, and the total count of effector memory CD4+ T cells was increased compared with both groups of single adjuvanted mice (**Figures 5E, F**).

**Figure 6:**
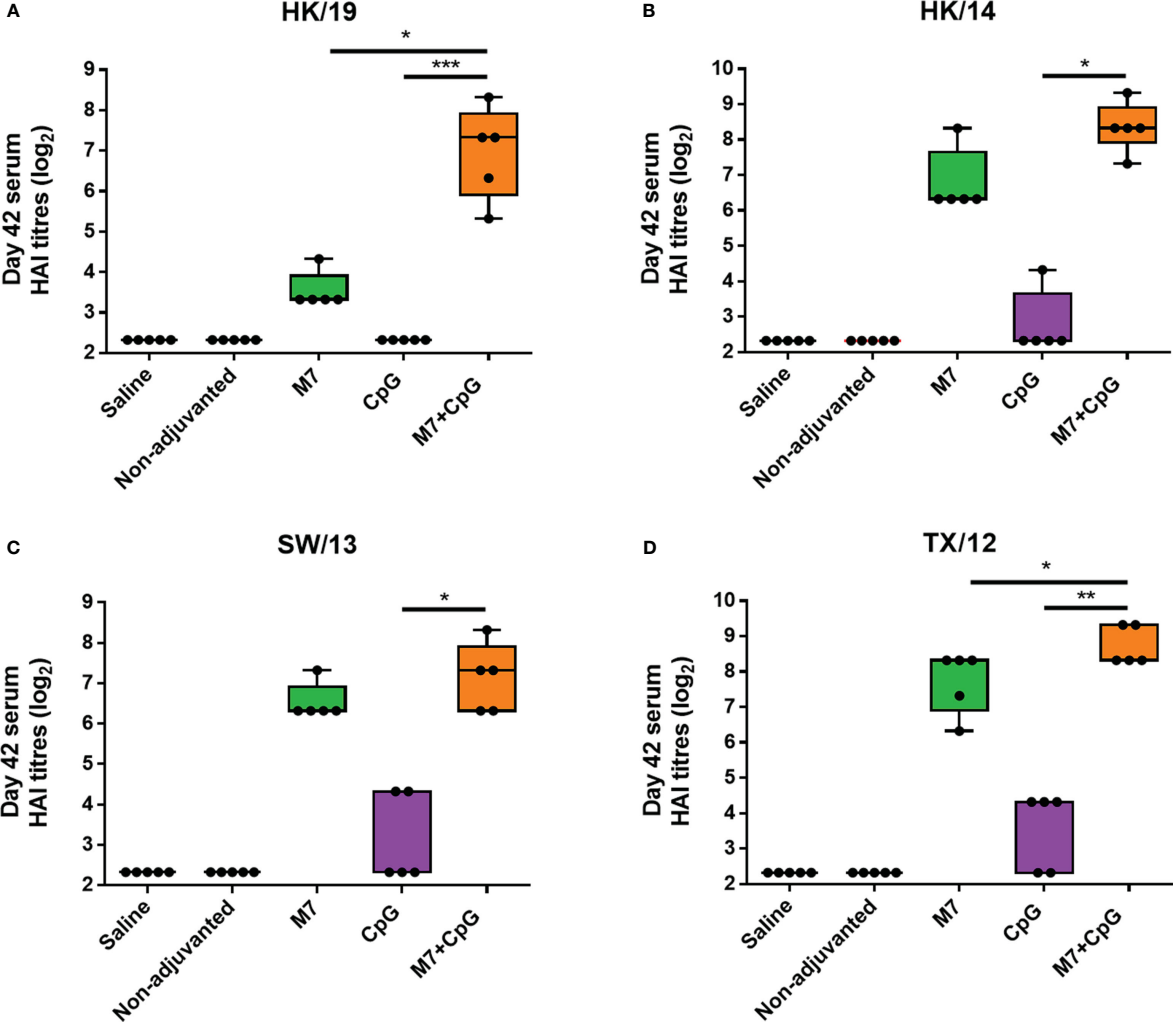
The M7-CpG NPs adjuvanted COBRA vaccine induced a broad HAI antibody response against influenza viruses. C57BL/6 mice were intranasally immunized with experimental groups on days 0, 21, and 35 and sera were collected on day 42. Day 42 sera from the different mouse groups were evaluated for HAI titers against the H3 influenza viruses **(A)** A/Hong Kong/2671/2019 (HK/19), **(B)** A/Hong Kong/4801/2014 (HK/14), **(C)** A/Switzerland/9715293/2013 (SW/13), and **(D)** A/Texas/50/2012 (TX/12). Data are presented as mean ± range (*n* = 5) (representative plots of two independent experiments). Significant differences were determined using a one-way ANOVA test with Tukey’s multiple comparison on the data shown (*p ≤ 0.05; **p ≤ 0.01; ***p ≤ 0.005).

**Figure 7:**
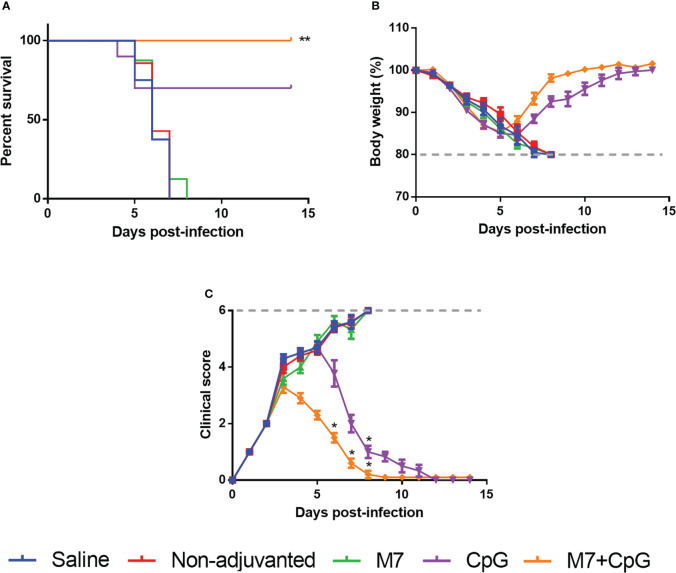
Vaccination with M7-CpG NPs and J4 COBRA protects against the H3 influenza virus challenge. C57BL/6 mice (*n* = 10) were intranasally immunized with saline, non-adjuvanted J4, M7 + J4, CpG + J4, and M7-CpG NPs + J4 on days 0, 21, and 35. Mice were challenged on day 56 with 1K PFUs of the A/Hong Kong/1/68 mouse-adapted virus. **(A)** Mice survival, **(B)** body weight, and **(C)** clinical score were evaluated for the different groups (*n* = 10). Data are presented as mean ± range. Significant differences were determined using a survival statistic and two-way ANOVA test with Tukey’s comparison against the saline group (*p ≤ 0.05; **p ≤ 0.01).

To assess functional viral neutralization, HAI assays against H3 influenza viruses [A/Hong Kong/2671/2019 (HK/19), A/Hong Kong/4801/2014 (HK/14), A/Switzerland/9715293/2013 (SW/13), and A/Texas/50/2012 (TX/12)] were conducted. Mice vaccinated with M7-CpG NPs had significantly higher (*p*-value ≤ 0.05) HAI titers against the four evaluated viruses than the CpG group, but compared with the M7 adjuvanted mice, higher HAI titers were found only for HK/19 and TX/12 viruses (**Figures 6A–D**). For protection studies, a strain predating J4 (mouse-adapted A/Hong Kong/1/1968) was used because of its ability to infect mice. Mice vaccinated with M7-CpG NPs achieved 100% survival against the virus, whereas the CpG-adjuvanted group had 70% survival upon challenge (**Figure 7A**). Mouse body weight was measured daily during the challenge, indicating that none of the tested vaccines could prevent loss of body weight after challenge. However, the M7-CpG NP-vaccinated group had a shorter weight recovery and better clinical outcome than CpG-vaccinated mice (**Figures 7B, C**).

## Discussion

### M7 and CpG form a nanoparticulate complex that can generate potent innate immune signaling

M7 and CpG stimulate inflammation through unique pathways. We therefore hypothesized that their combination could result in synergistic immune activation, with potential applications as a vaccine adjuvant. In an *in vitro* DC model, M7-CpG NPs (N/P ratio = 1) stimulated the most robust inflammatory cytokine secretion and the lowest cytotoxicity relative to M7 or CpG alone. Because M7 is a cationic peptide, we then speculated that CpG would form an ionic complex *via* interactions between M7 and the negatively charged DNA sugar-phosphate backbone. Consistent with this hypothesis, we observed the formation of self-assembled nanoparticulate structures when CpG and M7 were mixed at an N/P ratio = 1. Formation of NPs has been shown to allow for more efficient immune cell uptake compared with larger micron-sized particles (48, 49), so this may be beneficial for vaccine applications. Our data also indicate that M7-CpG NPs are stable when stored at 4°C and RT for at least 15 days, potentially allowing for easier storage requirements relative to many current vaccine formulations.

Combining adjuvants is a common strategy used to raise the immune response against co-immunized antigens (50, 51). CpG has been used for adjuvant combinations due to its potential to enhance the immune response against co-immunized viral antigens, even in aged mouse models (52, 53). Previously, it has been reported that CpG uptake is enhanced when combined with cationic peptides that act as transfection agents to enhance cell penetration (i.e., cell-penetrating peptides) (54). However, the addition of a cell-penetrating peptide alone is not sufficient to activate MCs, so the responses we observe are unique due to the inclusion of M7 (55). It is important to note that if M7 was simply acting as a cell-penetrating peptide, our studies with non-phagocytic lung epithelial LA-4 cells would likely illustrate similar cellular responses to DCs, but cytotoxicity was reduced in LA-4s. This could suggest that non-phagocytic cells do not efficiently take up M7-CpG NPs. This differential targeting represents an advantage because it can direct the adjuvant load to relevant innate immune cells, like DCs (56, 57).

The enhanced *in vitro* immune response we observed with DCs and MCs treated with M7-CpG NPs suggests that CpG can activate TLR9 in both cell types and potentiates the activation induced by M7 particularly in MCs, as previously described (58). Synergistic induction of IL-6 in MC by M7-CpG NPs is an important phenomenon in the generation of an optimal inflammatory environment in the immunization site, as it has been reported that IL-6 release by MCs increases the production of IgA by B cells (18). On the other hand, the degranulation levels of MCs stimulated with NPs were not statistically different in comparison with M7 alone, suggesting that CpG is not actively contributing to MC degranulation, which agrees with previous reports (59). However, not having overactivation of MCs by M7-CpG NPs can be a sign of safety, mainly because an impaired MC degranulation can lead to the development of hypersensitivity, allergy, or anaphylaxis (60).

### Vaccination with M7-CpG NPs and COBRA HA offers broad protection against influenza

MCs are abundant at sites of environmental interface, such as the pulmonary epithelia, and are major effector cells in the inflammatory response in the respiratory tract (61). For that reason, we decided to IN immunize mice with M7-CpG NPs and the COBRA HA H3 antigen J4. Mice were immunized in a prime–boost–boost schedule on days 0, 21, and 35, respectively. Serum and mucosal samples were obtained to evaluate the antibody response induced against J4.

Our *in vivo* data indicated the generation of J4-specific IgG and IgM titers in serum. This antibody response correlates with the enhanced innate immune response we observed *in vitro*. This increase in the antibody titers was also observed in the mucosal IgG and IgA titers in the respiratory tract. Additionally, the elevated J4-specific IgA titers in feces suggested the induction of systemic and local mucosal immune response. The generation of a synergistic serum antibody response has been described only for the co-immunization of CpG with cationic peptides administered in a parenteral route (62). Nonetheless, IN immunization of CpG can induce a mucosal immune response by itself (63, 64). Curiously, both J4-specific IgG1 and IgG2c antibody titers were higher in serum from mice vaccinated with NPs than mice vaccinated with the separated adjuvants (**Figure S2**), suggesting the presence of Th1 and Th2 responses. These results are promising as the presence of both T helper (Th) responses have been associated with an optimal immune response upon vaccination (65, 66); however, these data need to be corroborated by directly analyzing the T cells in future studies.

In addition to inducing a humoral response, we observed the formation of a robust cellular response following M7-CpG NP vaccination. Splenocytes from NP-treated mice exhibited enhanced IL-2 and IFN-γ secretion after antigen recall, indicating a significant Th1 response. A Th1 response is one of the main effector mechanisms of the immune system against intracellular pathogens like influenza virus infections and can facilitate recovery after infection (67, 68). Moreover, the increase in the number of memory CD4^+^ T cells in the dLN of vaccinated mice suggested a long-lasting immune response against the COBRA HA antigen. This characteristic can be achieved by the combination of CpG and poly-L-arginine or CpG alone (69, 70), as noticed in our results, but the number of memory T cells was tripled in mice vaccinated with M7-CpG NPs. Together, the resulting T-cell responses suggest that M7-CpG NPs induced a strong Th1 response and generated a memory T-cell response in the cervical dLN that can help to enhance protection in the immunization site.

Enhanced sera HAI titers against several H3 influenza viruses were observed in mice vaccinated IN with M7-CpG NPs. This enhanced response corroborates the broad reactivity (against different H3N2 influenza strains) of the COBRA antigen and the functionality of the adjuvant system. The HAI titers observed herein agree with results previously described for J4 antigen immunization in mice and humans (8, 71), which reinforces the promising future of COBRA-based vaccines. It is important to mention that the strains evaluated for HAI were constitutive strains used in the generation of COBRA J4; however, the older strain A/Hong Kong/1/68 H3N2 (HK/68) was not used.

To evaluate the potential for J4 to confer protection, mouse-adapted HK/68 virus was used to challenge vaccinated mice. Of note, HK/68 was not considered in the generation of J4 sequence (71). Nevertheless, COBRA HA has been previously evaluated against contemporary and future co-circulating isolates, resulting in the neutralization of these viruses (14 drift viruses and two viruses representing future strains) (72). In M7-CpG NP-vaccinated mice, 100% survival was achieved in the HK/68 challenge model, an event only achieved in this experimental group with the CpG group also illustrating considerable survival (70%). Challenge results show similarities with previous reports in which epitope-based vaccines induced protection in ferrets and J4-immunized mice had a total clearance of H3N2 in lungs (8, 73).

It is important to mention that mouse CpG was used in this work and the response can be different in human models. Moreover, safety is one of the main concerns regarding IN vaccination due to some adverse effects reported after the administration of live-attenuated or specific adjuvanted vaccines in clinical and preclinical assays (74, 75). However, even if further studies in humans are needed, CpG, M7, and other TLR ligands have been proven to be safe when given IN (14, 76, 77).

In summary, this study described a novel adjuvant system that not only induces a strong antibody and T-cell response in mice, but also provides 100% survival against an H3N2 challenge, as well as broad reactivity against several H3N2 viruses. This promising IN influenza vaccine can represent a strong candidate as a universal H3N2 influenza vaccine.

## Data availability statement

The raw data supporting the conclusions of this article will be made available by the authors, without undue reservation.

## Ethics statement

The animal study was reviewed and approved by the UNC Institutional Animal Care and Use Committee.

## Author contributions

LO-P, CB, DH, EP, JR, RS, and MC generated and analyzed the data. TR, SA, and HS worked on data analysis and discussion. EB and KA conceived the project and worked on data analysis and discussion. SS edited the manuscript for grammar. All authors contributed to the article and approved the submitted version.

## Supplementary material

Supplementary Figure 1 M7 and CpG form self-assembled NPs. TEM micrographs of **(A)** vehicle (PBS), **(B)** M7, **(C)** CpG and **(D)** the combination of M7 + CpG (N/P ratio = 1).

Supplementary Figure 2 M7-CpG NPs increase the serum antibody response against the COBRA influenza antigen J4. C57BL/6 mice were intranasally immunized with saline, non-adjuvanted J4, M7 + J4, CpG + J4 or M7-CpG NPs + J4 on day 0, 21 and 35. **(A)** J4-specific IgG1 and **(B)** IgG2c titers at day 42 post-immunization. Data are presented as mean ± range (n=5) (Representative plots of two independent experiments). Significant differences were determined using a one-way ANOVA test with Tukey´s multiple comparison (*p ≤ 0.05; **p ≤ 0.01).

Supplementary Figure 3 M7-CpG NPs enhance the T cell response against the COBRA influenza antigen J4. C57BL/6 mice were intranasally immunized with saline, non-adjuvanted J4, M7 + J4, CpG + J4 or M7-CpG NPs + J4 as previously described. Spleens and draining lymph nodes (dLNs) were harvested at day 42 post-immunization. **(A)** IL-2 and **(B)** IL-17 release was evaluated in supernatants from antigen-recalled splenocytes. **(C)** Representative dot plots of central memory (CM) and effector memory (EM) phenotype in CD4+ T cells from dLN (Dot plots comes from single, live CD3+ CD4+ cells). Data are presented as mean ± range (n=5) (Representative plots of two independent experiments). Significant differences were determined using a one-way ANOVA test with Tukey´s multiple comparison (*p ≤ 0.05; **p ≤ 0.01).

Supplementary Figure 4 CpG cytotoxicity on DC2.4 cells at 24 and 48 h post-stimulation. Dilutions of CpG used for N/P = 0.5 started at 32 µM, for N/P = 1 started at 16 µM, for N/P = 2 started at 8 µM, and for N/P = 3 started at 5 µM.

Supplementary Table 1 Table of statistical comparisons of . IL-6 data comparison between M7 alone and the different NP ratios (with p ≤ 0.05). ns, not significant.

Supplementary Table 2 M7-CpG NPs ELS Zeta potential and mobility.

Supplementary Table 3 M7-CpG NPs DLS diameter and PDI along different storage temperatures and storage periods.
