# Supplementary material for: Development of a broadly active influenza intranasal vaccine adjuvanted with self-assembled particles composed of mastoparan-7 and CpG
Source: Front Immunol. 2023 Mar 24;14:1103765. doi: 10.3389/fimmu.2023.1103765 (PMC10081679; doi:10.3389/fimmu.2023.1103765)
Supplement: Supplementary Figure 1 — M7 and CpG form self-assembled NPs. TEM micrographs of (A) vehicle (PBS), (B) M7, (C) CpG and (D) the combination of M7 + CpG (N/P ratio = 1). [file DataSheet_1.docx]

Supplementary Material

Development of a broadly active influenza intranasal vaccine adjuvanted with self-assembled particles composed of mastoparan-7 and CpG

Luis Ontiveros-Padilla^1^, Cole J. Batty^1^, Dylan A. Hendy^1^, Erik S. Pena^2^, John A. Roque III^1^, Rebeca T. Stiepel^1^, Michael A. Carlock^3,4^, Sean R. Simpson^1^, Ted M. Ross^3,4^, Soman N. Abraham^5^, Herman F. Staats^6,7^, Eric M. Bachelder^1^ and Kristy M. Ainslie^1,2,8^*

^1^Division of Pharmacoengineering and Molecular Pharmaceutics, Eshelman School of Pharmacy, University of North Carolina at Chapel Hill, Chapel Hill, NC, United States, ^2^Department of Biomedical Engineering, NC State/UNC, Chapel Hill, NC, United States, ^3^Florida Research and Innovation Center, Port Saint, Cleveland Clinic Florida, Port St. Lucie, FL, United States, ^4^Center for Vaccines and Immunology, College of Veterinary Medicine, University of Georgia, Athens, GA, United States, ^5^Departments of Pathology, Molecular Genetics and Microbiology and Immunology, Duke University School of Medicine, Durham, NC, United States, ^6^Department of Pathology, School of Medicine, Duke University, Durham, NC, United States, ^7^Duke Human Vaccines Institute, School of Medicine, Duke University, Durham, NC, United States, ^8^Department of Microbiology and Immunology, School of Medicine, University of North Carolina at Chapel Hill, Chapel Hill, NC, United States.

*** Correspondence:** Corresponding Author: ainsliek@email.unc.edu

# Supplementary Figures and Tables

## Supplementary Figures

**Figure S1. M7 and CpG form self-assembled NPs.** TEM micrographs of **(A)** vehicle (PBS), **(B)** M7, 718 **(C)** CpG and **(D)** the combination of M7 + CpG (N/P ratio = 1).


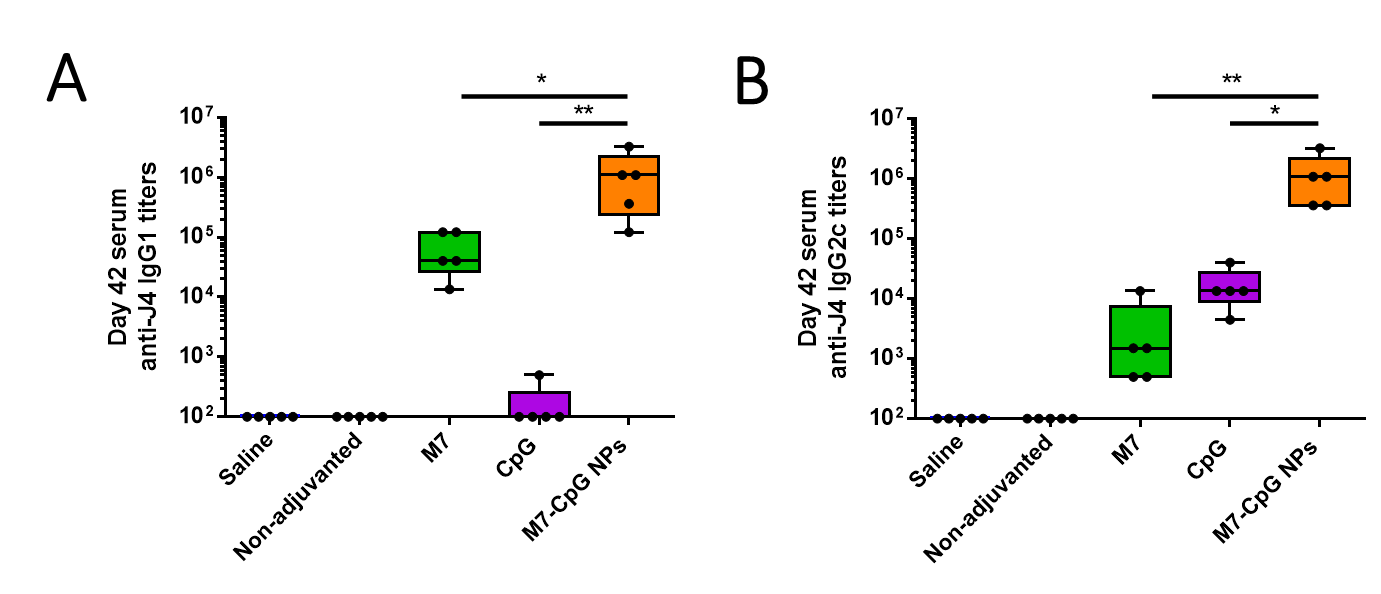


**Figure S2. M7-CpG NPs increase the serum antibody response against the COBRA influenza antigen J4.** C57BL/6 mice were intranasally immunized with saline, non-adjuvanted J4, M7 + J4, CpG + J4 or M7-CpG NPs + J4 on day 0, 21 and 35. **(A)** J4-specific IgG1 and **(B)** IgG2c titers at day 42 post-immunization. Data is presented as mean ± range (n=5) (Representative plots of 2 independent experiments). Significant differences were determined using a one-way ANOVA test with Tukey´s multiple comparison (*p≤0.05; **p≤0.01).


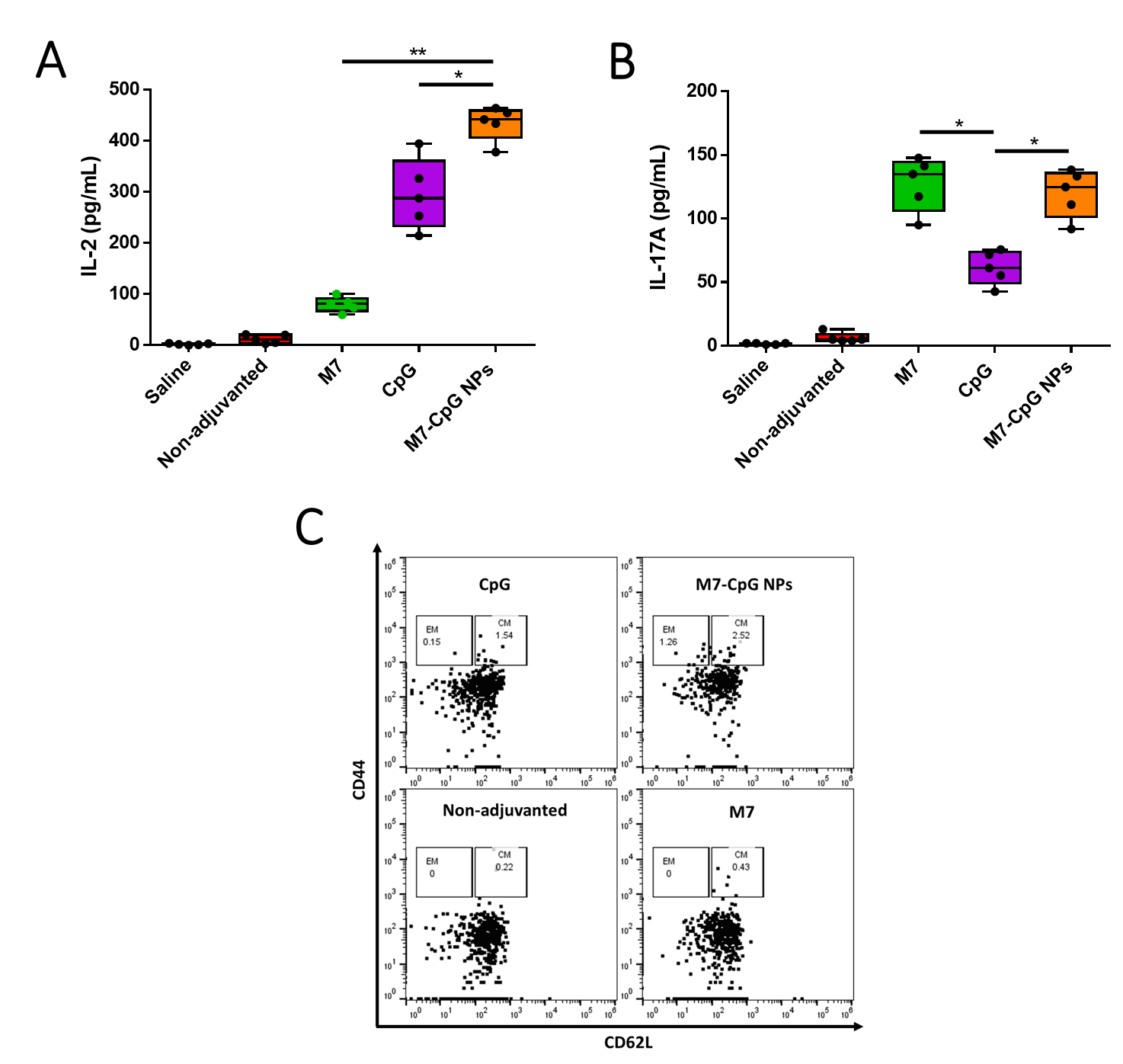


**Figure S3. M7-CpG NPs enhance the T cell response against the COBRA influenza antigen J4.** C57BL/6 mice were intranasally immunized with saline, non-adjuvanted J4, M7 + J4, CpG + J4 or M7-CpG NPs + J4 as previously described. Spleens and draining lymph nodes (dLNs) were harvested at day 42 post-immunization. **(A)** IL-2 and **(B)** IL-17 release was evaluated in supernatants from antigen-recalled splenocytes. **(C)** Representative dot plots of central memory (CM) and effector memory (EM) phenotype in CD4+ T cells from dLN (Dot plots comes from single, live CD3+ CD4+ cells). Data is presented as mean ± range (n=5) (Representative plots of 2 independent experiments). Significant differences were determined using a one-way ANOVA test with Tukey´s multiple comparison (*p≤0.05; **p≤0.01).

**Figure S4. CpG cytotoxicity on DC2.4 cells at 24 and 48 h post-stimulation.** Dilutions of CpG used for N/P = 0.5 started at 32 µM, for N/P = 1 started at 16 µM, for N/P = 2 started at 8 µM and for N/P = 3 started at 5 µM.

| **M7 concentration**  **(µM)** | **Tukey´s multiple comparisons test** | **Mean Diff.** | **95% CI of diff.** | **Significant?** | **Summary** |
| --- | --- | --- | --- | --- | --- |
| 35 | M7 + CpG N/P=0.5 vs. M7 | -221.8 | -345.4 to -98.13 | Yes | ** |
|  | M7 + CpG N/P=1 vs. M7 | 185.8 | 62.21 to 309.5 | Yes | ** |
|  | M7 + CpG N/P=2 vs. M7 | 104.0 | -19.62 to 227.6 | No | ns |
|  | M7 + CpG N/P=3 vs. M7 | 73.73 | -49.90 to 197.4 | No | ns |
| \| 70 \| \| --- \| | M7 + CpG N/P=0.5 vs. M7 | -131.4 | -313.8 to 50.97 | No | ns |
|  | M7 + CpG N/P=1 vs. M7 | 183.4 | 1.002 to 365.8 | Yes | * |
|  | M7 + CpG N/P=2 vs. M7 | 252.9 | 70.49 to 435.2 | Yes | ** |
|  | M7 + CpG N/P=3 vs. M7 | 717.2 | 534.8 to 899.6 | Yes | **** |
| \| 140 \| \| --- \| | M7 + CpG N/P=0.5 vs. M7 | 181.8 | -12.77 to 376.5 | No | ns |
|  | M7 + CpG N/P=1 vs. M7 | 203.3 | 8.675 to 397.9 | Yes | * |
|  | M7 + CpG N/P=2 vs. M7 | 706.4 | 511.8 to 901.0 | Yes | **** |
|  | M7 + CpG N/P=3 vs. M7 | 1144 | 949.0 to 1338 | Yes | **** |

**Table S1. Table of statistical comparisons of Figure 1D.** IL-6 data comparison between M7 alone and the different NP ratios (with p≤0.05). NS=not significant.

| **Condition** | **ELS Zeta**  **potential**  **(mV)** | **ELS Zeta potential SD (+/-)** | **ELS mobility**  **(μ/s) / (V/cm)** | **ELS mobility SD (+/-)** |
| --- | --- | --- | --- | --- |
| **PBS** | 0.00 | 0.00 | 0.00 | 0.00 |
| **M7** | 0.00 | 0.00 | 0.00 | 0.00 |
| **CpG** | 0.00 | 0.00 | 0.00 | 0.00 |
| **M7+CpG N/P=1** | -32.29 | 5.87 | -2.54 | 0.18 |

**Table S2. M7-CpG NPs ELS Zeta potential and mobility.**

| **Storage**  **Temperature**  **(°C)** | **Storage**  **time**  **(d)** | **Average diameter**  **(nm)** | **Average**  **diameter**  **SD (+/-)** | **PDI** | **PDI**  **SD (+/-)** |
| --- | --- | --- | --- | --- | --- |
| 20 | 0 | 228.97 | 12.54 | 0.233 | 0.02 |
|  | 2 | 220.25 | 10.08 | 0.214 | 0.02 |
|  | 5 | 241.58 | 15.25 | 0.275 | 0.04 |
|  | 10 | 297.72 | 15.18 | 0.207 | 0.03 |
|  | 15 | 266.82 | 12.83 | 0.235 | 0.05 |
| 4 | 0 | 222.39 | 10.45 | 0.210 | 0.04 |
|  | 2 | 237.22 | 11.43 | 0.201 | 0.03 |
|  | 5 | 283.86 | 14.68 | 0.225 | 0.08 |
|  | 10 | 360.88 | 12.44 | 0.231 | 0.02 |
|  | 15 | 331.20 | 17.86 | 0.268 | 0.05 |
| -20 | 0 | 221.38 | 12.57 | 0.228 | 0.03 |
|  | 1 | 412.65 | 39.31 | 0.314 | 0.09 |
|  | 2 | 481.05 | 34.04 | 0.325 | 0.07 |
|  | 3 | 560.53 | 31.77 | 0.318 | 0.05 |
|  | 5 | 749.84 | 28.56 | 0.322 | 0.08 |

**Table S3. M7-CpG NPs DLS diameter and PDI along different storage temperatures and storage periods.**
